# Evaluation of percutaneous unilateral trans-femoral implantation of side-hole port-catheter system with coil only fixed-catheter-tip for hepatic arterial infusion chemotherapy

**DOI:** 10.1186/s40644-019-0202-z

**Published:** 2019-03-18

**Authors:** Jungang Hu, Xu Zhu, Xiaodong Wang, Guang Cao, Xiao Wang, Renjie Yang

**Affiliations:** 1 Department of Interventional Radiology, Peking University Cancer Hospital & Institute, Key laboratory of Carcinogenesis and Translational Research (Ministry of Education), 52 Fucheng Road, Haidian District, Beijing, 100142 China; 2 Department of Epidemiology and Biostatistics, Peking University Sixth Hospital, Beijing, 100191 China

**Keywords:** Fixed catheter tip, Hepatic arterial infusion chemotherapy, Femoral artery, Hepatic tumor, Hepatic metastasis, Interventional oncology

## Abstract

**Background:**

The technique for arterial infusion chemotherapy (HAIC) is not standardized which limits its widely application. The aim of this study was to evaluate the long-term functionality and complications of port-catheter system using percutaneous unilateral trans-femoral implantation with coil only fixed-catheter-tip method.

**Methods:**

From January 2013 to January 2017, 205 consecutive patients (138 men; aged 28–88 years; mean, 59.1 ± 11.2 years) with unresectable malignant liver tumors underwent percutaneous implantation of side-hole infusion port-catheter into hepatic artery using coil only fixed-catheter-tip method via the unilateral femoral artery. Technical success, procedure time, duration of port functionality, and complications of port dysfunction were investigated.

**Results:**

Implantation technical success was 98.5% and the procedure time was 59.1 ± 10.2 min. Predictable functionality of the port-catheter system at 6-, 12-, and 24 months were 97.5, 89.9, 70.5%, respectively. Complications of port irreversible dysfunction were hepatic artery obstruction (4.0%), catheter occlusion (3.5%), and catheter dislocation (0.5%). Median 5 HAIC cycles (range: 1–14 cycles) were received via port.

**Conclusion:**

Percutaneous unilateral trans-femoral implantation of a side-hole port-catheter with coils only fixed-catheter-tip method is a simple and feasible interventional technique for HAIC which offers long-term functionality.

## Background

Hepatic artery infusion chemotherapy (HAIC) has been an encouraging method for patients with unresectable malignant liver tumors, such as colorectal cancer liver metastasis, cholangiocarcinoma, with higher local tumor response rate and better survival benefit than systemic chemotherapy taking advantage of the fact that HAIC achieve much higher local chemotherapeutic agent concentration comparing with systemic chemotherapy [1–5]. Although the systemic chemotherapy with gemcitabine plus cisplatinum was deemed as standard treatment for advanced biliary tract cancer due to ABC-02 trail, but survival benefit was limited [6]. In a recent meta-analysis, for patients with unresectable intrahepatic cholangiocarcinoma (ICC), HAIC offered the best outcomes in terms of tumor response and survival compared to other arterial directed therapies including transarterial chemoembolization (TACE), drug-eluting bead TACE (DEB-TACE), and Yttrium-90 radioembolization (^90^Y) [7]. A recently published prospective phase II study of HAIC for advanced perihilar cholangiocarcinoma also showed promising result with overall survival of 20.5 months [4].

To facilitate long-term administration of anticancer agents a permanent arterial port-catheter system has been implanted to allow repetitive HAIC [8–12]. However, techniques of port implantation for HAIC were not made in consensus which limit its widely application.

Traditionally, port-catheter system placement is accomplished via surgical laparotomy under general anesthesia. However, recent advances in minimally invasive techniques allow percutaneous placement of catheter-port systems under local anesthesia for HAIC [13]. Among methods of implantation by interventional techniques [11, 14–17], implantation with the fixed-catheter-tip method prevents catheter dislocation and hepatic artery obstruction which was mostly used in Japan [17–19]. But for fixed catheter-tip method of port implantation, a lot of intra-procedural technical points were not made in consensus such as unilateral or bilateral arterial access, arterial access between femoral, subclavian, hypogastric and brachial arteries, catheter tip fixation with glue or not, and also the catheter system [17, 20, 21]. Percutaneous unilateral trans-femoral implantation of a side-hole port-catheter with coils only fixed-catheter-tip method was an easily and widely performed procedure in our center. The aim of this single center study was to evaluate the experience of 205 consecutive cases of this technique with regard to long-term functionality and complications data.

## Methods

### Subjects

From January 2013 to January 2017, 205 consecutive patients who were selected for HAIC with port catheter system in our institution were retrospectively analyzed. Patient eligibility criteria of port implantation for HAIC: unresectable hepatocellular carcinoma (HCC) with portal vein tumor thrombosis; unresectable intrahepatic or perihilar cholangiocarcinoma; unresectable liver metastasis from colorectal cancer and other gastrointestinal cancers usually after failure of first or second line systemic chemotherapy. Small extrahepatic disease confirmed by radiologic examination or intraoperative findings was not considered an absolute contraindication for catheter placement if the liver was the predominant site of disease. Other eligibility criteria for implanting port-catheter system were as follows: Eastern Cooperative Oncology Group (ECOG) performance status ≤2; Child-Pugh classification A or B; albumin > 2.5 g/dl; alanine aminotransferase and aspartate aminotransferase < 5 times the upper normal limit; total serum bilirubin < 3.0 mg/dl; serum creatinine < 2.0 mg/dl; platelet count > 50,000/mm3 and international normalized ratio (INR) ≤1.5. Of the 205 patients, seventy patients had perihilar cholangiocarcinoma, 55 had primary liver cancer (ICC, 39; HCC, 15; mixed type, 1), 80 had metastatic liver cancer originating from colorectal cancer (*n* = 51), gastric cancer (*n* = 7), gallbladder cancer (*n* = 18), esophageal cancer (*n* = 2), and pancreas cancer (n = 2). Patient clinical characteristics for those who underwent percutaneous implantation of the port-catheter appear in Table 1. A waiver of authorization was obtained from the local ethics committee for this retrospective study. Written informed consent was obtained from each patient or their family members before port-catheter implantation.

**Table 1:** Clinical characteristics of patients underwent percutaneous port-catheter system Implantation

Characteristics	Numbers
Sex	
Male	138 (67.3%)
Female	67 (32.7%)
Age (y)	59.1 ± 11.2
Performance status	
0	149 (72.7%)
1	43 (21.0%)
2	13 (6.3%)
Perihilar cholangiocarcinoma	70 (34.1%)
Primary liver cancer	55 (26.8%)
Hepatocellular carcinoma	15 (7.3%)
Intrahepatic cholangiocarcinoma	39 (19.0%)
Mixed type	1 (0.5%)
Metastatic liver cancer	80 (39.0%)
Colorectal cancer	51 (24.9%)
Gallbladder cancer	18 (8.8%)
Gastric cancer	7 (3.4%)
Esophageal cancer	2 (1.0%)
Pancreatic cancer	2 (1.0%)

### Implantation of the port-catheter system

**Fig.1:**
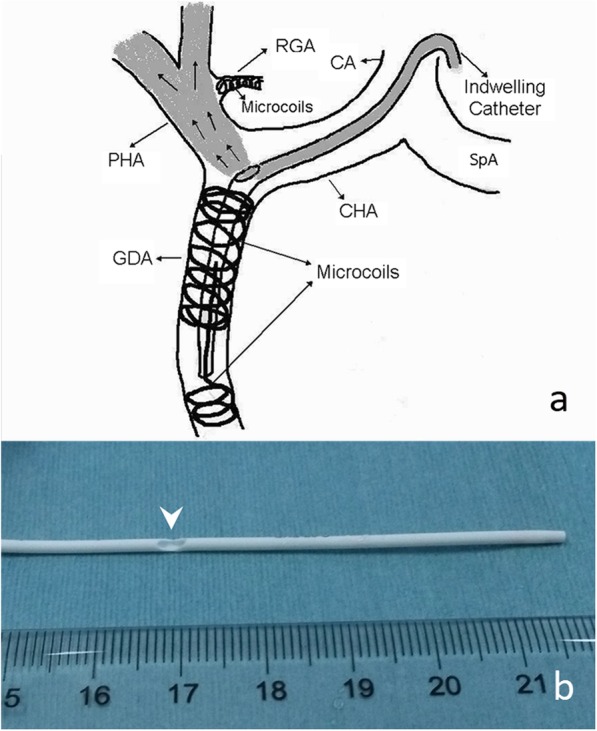
**a**. Schematic of percutaneous implantation of side-hole port-catheter system with coil-fixed-catheter-tip. CA = coeliac axis; CHA = common hepatic artery; GDA = gastroduodenal artery; PHA = proper hepatic artery; RGA = right gastric artery; SpA = splenic artery. **b**. Distal part of the indwelling catheter with side hole (white arrowhead) 5 cm to catheter tip

**Fig. 2:**
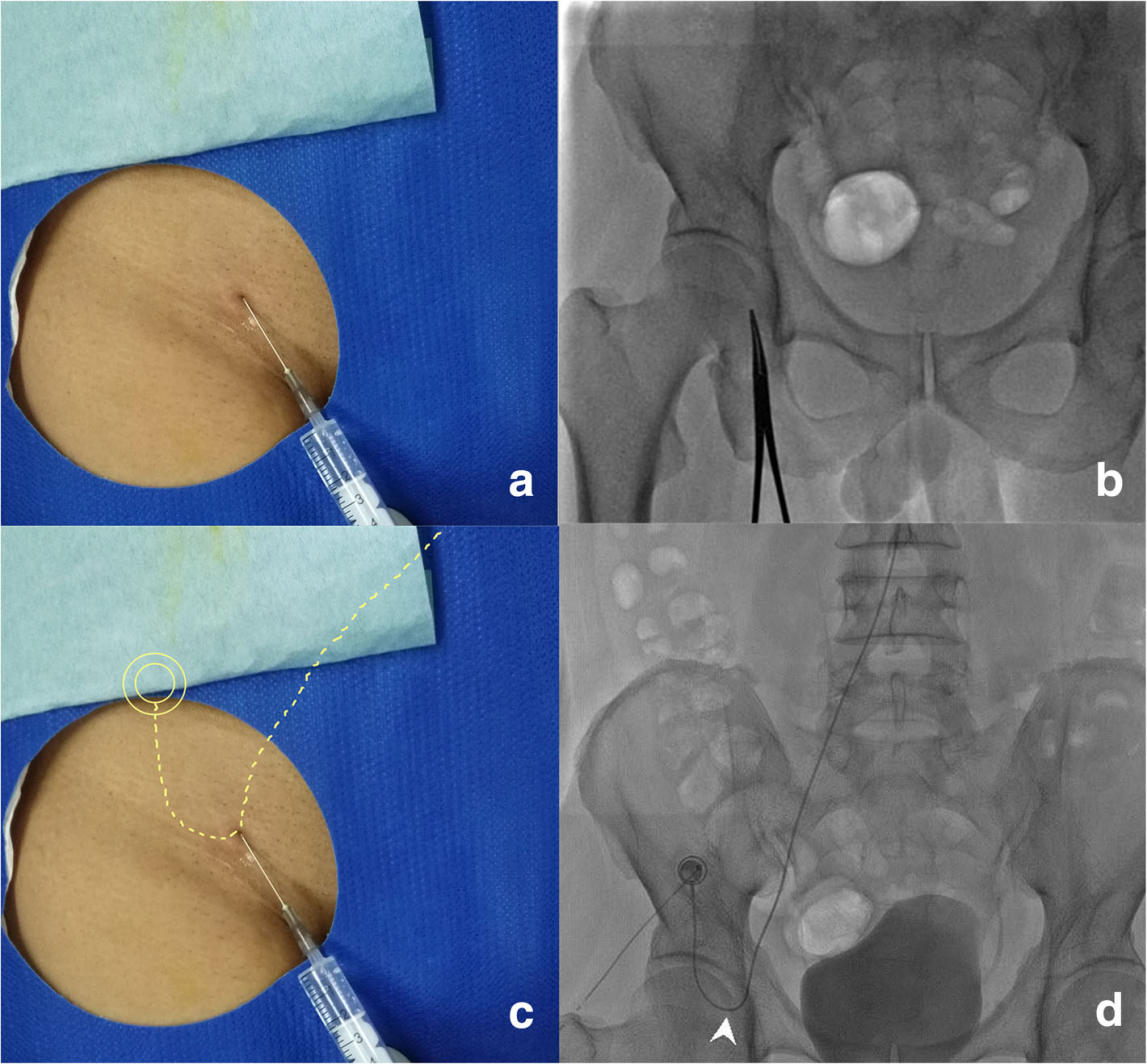
Location of skin access site and implanted port. **a**. Needle tip indicates skin incision access site ~ 1 cm above inguinal skin fold; **b**. Forcep head tip (same site as needle tip) indicates skin incision site with fluoroscopic image. **c**, **d**. Implanted port situated 2 cm medial to the antero-superior iliac crest, the lowest loop of the indwelling catheter (yellow dotted line) located above the inguinal site (**c**), and at the upper half portion of femur head (white arrowhead) on fluoroscopic image

**Fig. 3:**
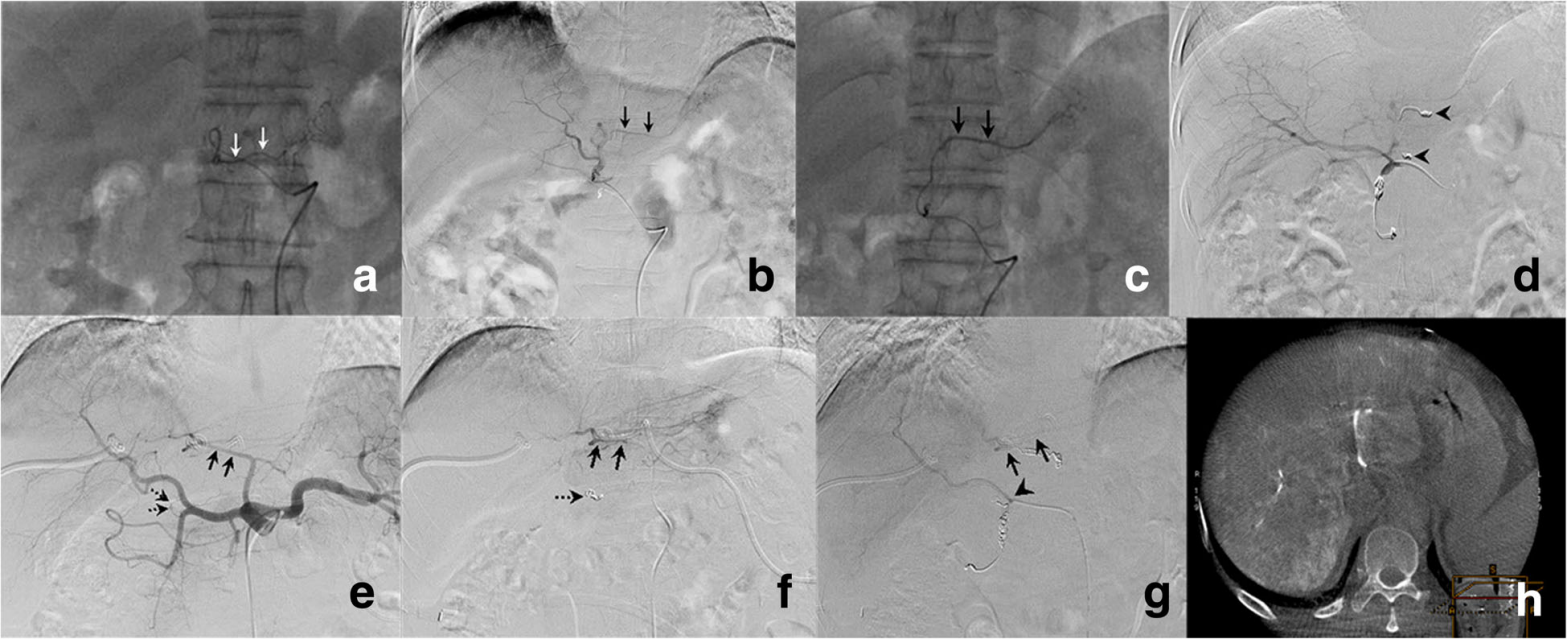
Embolization of extrahepatic arteries and Hepatic artery redistribution embolization. **a**. Selective right gastric arteriogram from left hepatic artery shows right gastric artery (white arrows), embolized with microcoils; **b**. Left hepatic arteriogram showed accessory left gastric artery (black arrows) arising from left hepatic artery; **c**. Accessory left gastric artery (black arrows) was selectively catheterized and embolized with coils; **d**. Proper hepatic arteriogram shows whole hepatic artery without extrahepatic supplies. Black arrowheads refer to coils embolized in the accessory left gastric artery and right gastric artery. **e**. Celiac arteriogram indicates replaced left hepatic artery (black arrows) arising from left gastric artery. Black dotted arrows indicate right gastric artery. **f**. Replaced left hepatic artery (black arrows) selectively catheterized and embolized with microcoils. Right gastric artery was embolized with microcoils (black dotted arrow). **g**. Proper hepatic arteriogram from side hole (black arrowhead) of indwelling catheter shows whole hepatic arterial flow including right hepatic artery and the redistributed left hepatic artery (black arrows). **h**. CBCT-proper hepatic arteriogram shows contrast enhancement of the entire liver

Percutaneous implantation of the side-hole port-catheter system (Fig. 1a) was performed by two interventional radiologists with 15 and 18 years experience in vascular interventional radiology. Immediately before the procedure, patients were given local anesthesia with 1% lidocaine. Skin incision one centimeter above the inguinal skin fold was made to access the femoral artery (Fig. 2a-d). A 5F introducer was inserted in the femoral artery without a sheath to avoid leakage at the puncture site. Then a 5F Yashiro catheter (TERUMO, Japan) was placed into the femoral artery through a 0.035-in. guide wire. Superior mesenteric and celiac angiographies were obtained to assess arterial supply to the liver. If extrahepatic arteries such as the right gastric artery, accessory left gastric artery and supraduodenal arteries were identified, they were embolized with 0.018-in. micro-coils (Cook, USA, or Boston Scientific, USA) after catheterization with a 2.7F microcatheter (TERUMO, Japan) (Fig. 3a-d). When an aberrant hepatic artery was encountered, hepatic arterial blood flow was redistributed using 0.018-in. micro-coils to convert multiple hepatic arteries into a single arterial blood supply (Fig. 3e-h).

**Fig.4:**
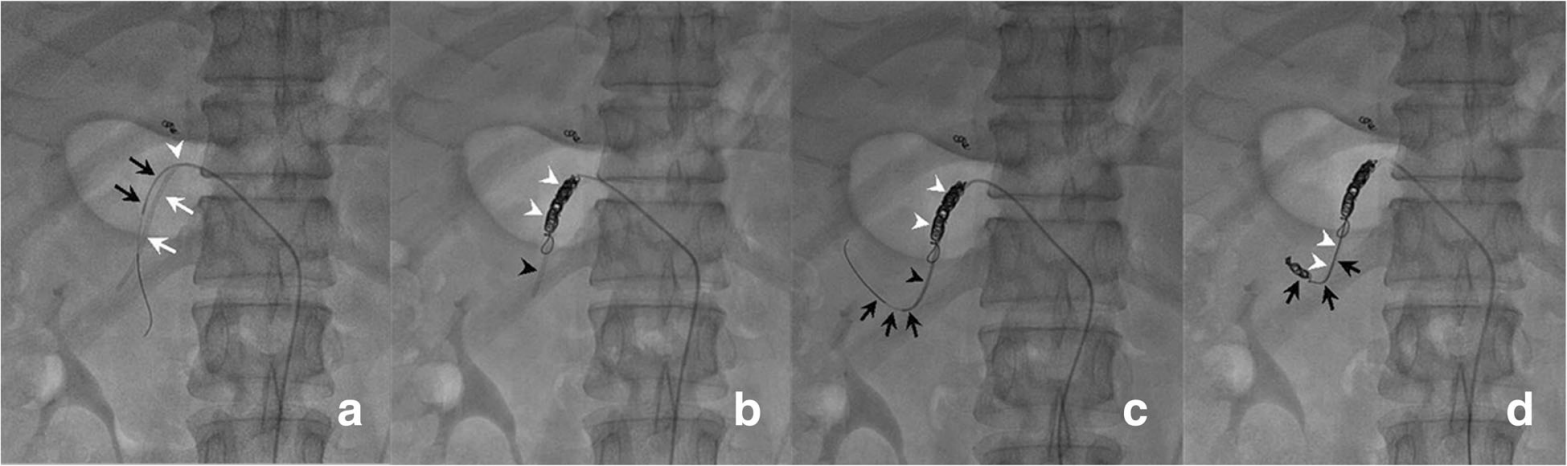
**a**. Fluoroscopic image shows microcatheter (black arrows) inserted coaxially through side hole (white arrowhead) of indwelling catheter to gastroduodenal artery outside distal part of indwelling catheter (white arrows); **b**. Fluoroscopic image shows that tip of indwelling catheter (black arrowhead) is fixed to gastroduodenal artery with microcoils (white arrowheads); **c**. Fluoroscopic image shows that microcatheter (black arrows) coaxially passed through inside lumen of indwelling catheter tip (black arrowhead); **d**. Microcoil (black arrows) embolized to occlude the inside lumen of the distal tip of the indwelling catheter (white arrowheads)

Then a long, tapered 5F catheter (Celsite 5F Implantofix 4,438,663 or Celsite 5F PSU ST305C 4,436,962, B. BRAUN MEDICAL, France) was used as an indwelling catheter and inserted over a 0.035-in. guidewire through the right femoral artery into the gastroduodenal artery by way of the celiac trunk. The tip of the indwelling catheter was advanced over the exchange guide wire 5 cm into the gastroduodenal artery with the tip usually in the right gastroepiploic artery. A side hole was manually created in the indwelling catheter (5 cm from the tip) before its insertion (Fig. 1b). The position of the side hole was adjusted to locate at the final portion of the common hepatic artery just before the gastroduodenal artery arises, and the catheter tip was “fixed” [22] to the gastroduodenal artery with 0.018-in. coils. Avoiding puncturing the contralateral femoral artery, this embolization was performed using coaxial technique with a 2.7F microcatheter (TERUMO, Japan) coaxially through indwelling catheter and exited by way of its side hole to reach the gastroduodenal artery. Micro-coils were then inserted into gastroduodenal artery around the tip of the side-hole catheter through the microcatheter, and the distal tip of the catheter was fixed in the gastroduodenal artery. The inside lumen of the distal tip of the indwelling catheter was occluded with a microcoil through a microcatheter that was advanced coaxially the indwelling catheter beyond the side hole (Fig. 4a-d).

**Fig. 5:**
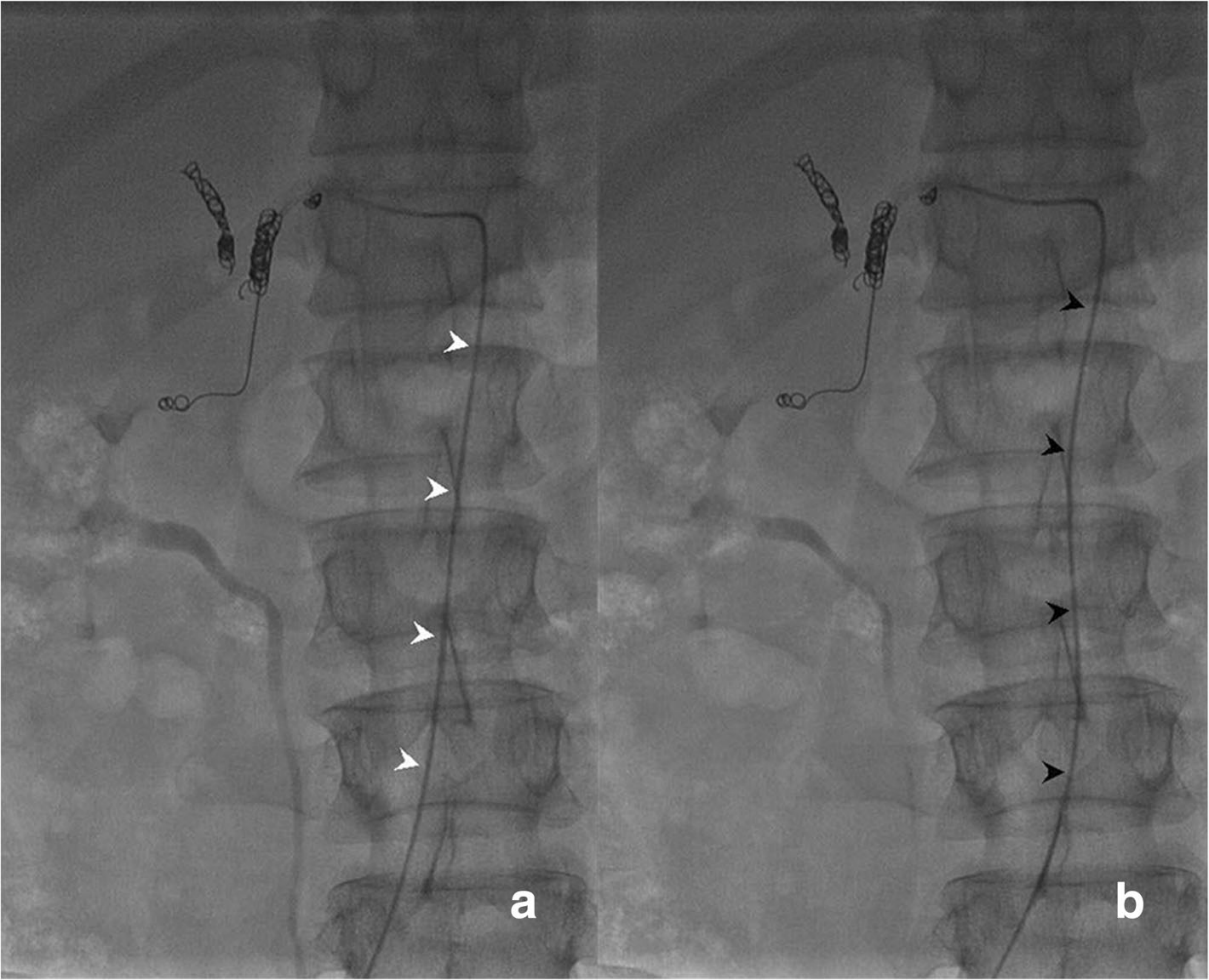
Pushing indwelling catheter careful 1-2 cm within femoral artery to obtain sufficient curve (**b** black arrowheads) of the catheter from a straight line (**a** white arrowheads) in the aort

Finally, the catheter was advanced 1–2 cm within the femoral artery to obtain a comfortable curve of the catheter and lack catheter tension within the aorta (Fig. 5a-b). The proximal end of the indwelling catheter was cut and connected through a subcutaneous tunnel to a subcutaneously implanted port through a skin incision performed 2 cm medial to the antero-superior iliac crest (Fig. 2c, d). After connecting the catheter to the port, contrast medium was injected into the port using a 19-gauge Huber needle. Hepatic arteriogram (volume 6-8ml, rate 1ml/s) and or cone-beam computer tomograpy (CBCT) hepatic arteriograms (volume 15ml, rate 1ml/s, scanning delay 10s) through ports were performed. Port-catheter implantation was considered technically successful if the contrast medium injected through the port entirely opacified the liver without extrahepatic perfusion.

The port pocket was flushed with antibiotic solution and sutured aseptically and 2000 IU of heparin was inserted into the port. To prevent thrombosis of the port-catheter system, the port was flushed with heparinized saline (2000 IU in 2 ml) after each administration of chemotherapeutic drugs.

### Port-catheter system follow-up

Before each infusion, patients had digital subtraction angiography (DSA) of hepatic arterial circulation with injection of contrast (volume 6-8ml, rate 1ml/s) via the port system to confirm port patency and catheter tip. After each routine angiography via the catheter and after HAIC, the catheter was flushed with 2000 IU heparin (2 ml solution in 1000 IU/ ml). This angiographic study was performed every 3 months during port-catheter system maintenance.

### Hepatic arterial infusion chemotherapy

The most commonly used regimen for HAIC is oxaliplation + 5-fluorouracil which consisted of infusions of oxaliplatin (35–40 mg/m^2^ for 2 h), followed by fluorouracil (5-FU) (600–800 mg/m^2^ for 22 h) on day 1–3 every 3–4 weeks [4]. For each cycle, leucovorin calcium 200 mg/m^2^ was administered for 2 h beginning of the 5-FU infusion. Other HAIC regimens include irinotecan+ 5-FU and gemcitabine + nedaplatin. Maximally 6 cycles of HAIC were used for patients without disease progression during treatments. Dose modifications were defined per protocol. Modifications and delays were allowed for hematologic toxicity, abnormal liver and renal function, nausea, vomiting, and peripheral neuropathy. Maintenance was continued with oral capecitabine 1000 mg/m^2^ twice daily on days 1–14 every 21 days for most patients who achieved liver disease control after a 5-FU-contained HAIC regimen. HAIC will be resumed for cases of liver tumor progress during maintenance or follow up. HAIC treatment was discontinued because of maintenance treatment without disease progression, disease progression during HAIC, port dysfunction, patient or clinician choice, or unacceptable toxic effects. Patients abdominal image follow-up (contrast enhanced computer tomography/ magnetic resonance imaging) were performed every 2 cycles of HAIC, and then 3 months during maintenance treatment or the follow-up period. Because selection criteria for HAIC were not homogeneous, overall survival was not evaluated in this study.

### Port-catheter system functionality evaluation and salvage treatment for dysfunction

Functionality of the port-catheter systems were evaluated by two experienced interventional radiologist (18 and 15 years experience in liver intervention) in common to make consensus. Functionality refers to the normal flow of port catheter system without contrast injection resistance, and normal hepatic artery demonstration without obvious extrahepatic feeding arteries shown on angiogram via the port system. Any conditions which cannot meet above criteria were defined as dysfunction which includes migration of the catheter (side hole), catheter occlusion, or hepatic artery occlusion. Infusions were discontinued in cases of dysfunction of port system.

Salvage treatments for dysfunction: In the case of hepatic artery occlusion, 250,000 IU of urokinase in 50 ml saline solution was infused through the arterial port catheter over 2 h. In cases of port catheter occlusion, 250,000 IU of urokinase in 10 ml saline solution was used to flush the catheter with a 1 ml syringe. If not successful, these high concentration urokinase saline solution remained in the port catheter for 24 h for reexamination. If the catheter migrated to the celiac artery, the left gastric and splenic arteries were embolized with coils to redistribute all the blood flow of celiac artery only into hepatic artery. Angiography via the port system was performed again to re-evaluate the functionality after salvage treatment. HAIC through the port-catheter system was contraindicated for irreversible dysfunction.

### Parameters investigated

The following factors were investigated: technical success rate of port-catheter placement, the time required for the procedure, 6-, 12-, 24 months port catheter system functionality rate, complications that closely correlated with port system placement and complications which caused port system dysfunction. Port catheter system functionality refers to the normal function and reversible dysfunction.

## Results

202/205 patients were successfully implanted port system for HAIC with this technique. The intra-procedure complications and other data for this procedure are listed in Table 2. No bleeding, infection, and perioperative deaths occurred. Embolization was performed on 17 replaced hepatic arteries and 22 accessory hepatic arteries and 69 extrahepatic arteries.

**Table 2:** Data of the port implantation procedure

Procedures	Numbers
Success	202 (98.5%)
Time for the procedure	59.1 ± 10.2 min (45–107 min)
Failure	3 (1.5%)
Reason of failure	
Stenosis of celiac trunk	1 (0.5%)
Tortuosity of hepatic artery	1 (0.5%)
Catheter dislocation	1 (0.5%)
Introprocedure complications	
Migration of micro-coils into hepatic artery	4 (2.0%)
Infection	0
Bleeding	0
Embolization of replaced hepatic arteries	17 (8.4%)
Left hepatic artery	3 (1.5%)
Right hepatic arteries	14 (6.9%)
Embolization of accessory hepatic arteries	22 (10.9%)
Left hepatic artery	17 (8.4%)
Right hepatic arteries	5 (2.5%)
Embolization of extrahepatic arteries	69 (34.2%)
Right gastric artery	60 (29.7%)
Accessory left gastric artery	6 (3.0%)
Supraduodenal artery	3 (1.5%)

### Port-catheter system functionality and result by salvage treatment

Median follow-up time was 7.2 months (1.0–40.0 months). Table 3 depicts port system complications and results of salvage treatments. For 11 cases of hepatic artery occlusion, thrombolysis with urokinase via a port was performed for 6 patients. Hepatic arteries were recanalized for 3 cases. For 8 patients with permanent hepatic artery occlusion, HAIC treatment was stopped. For 8 patients with port-catheter occlusion, thrombolysis with urokinase via port was performed for 3 cases and port-catheters were recanalized for 1 patient after 24h of re-examination. For the 7 cases with irreversible port catheter occlusion, HAIC continued with a temporary implanted catheter from the other femoral artery. For 2 patients with catheter dislocation, left gastric artery and splenic artery were embolized with coils to eliminate the extrahepatic perfusion for one patient.

**Table 3:** Catheter Complications and Salvage Treatments

Complications	Patients	Time to complication diagnosis (months)	Salvage treatments	Result of salvage treatment
Catheter dysfunction				
Hepatic arterial occlusion	11 (5.4%)	0.7–27.5 Median 6.3	Thrombolysis via port in 6 patients	3 Successful/3 Failure
Occlusion of catheter	8 (4.0%)	9.3–29.5 Median 19.1	Thrombolysis via port in 3 patients	1 Successful/2 Failure
Catheter dislocation	2 (1.0%)	1.3 and 3.2	Blood flow redistribution embolization with coils in 1 patient	1 Successful
Catheter irreversible dysfunction				
Hepatic arterial occlusion	8 (4.0%)	2.9–28.6 Median 12.7		
Catheter occlusion	7 (3.5%)	9.3–29.5 Median21.1		
Catheter dislocation	1 (0.5%)	1.3		

In 3 patients with intra-procedural microcoils migration to proper hepatic artery, hepatic artery obstruction occurred at 1.3, 2.9 and 7.3 months, in which one recanalized after thrombolysis treatment. One patient suffered microcoil migration to the right hepatic artery, which did not result in thrombotic hepatic artery occlusion but vessel stenosis.

**Fig. 6:**
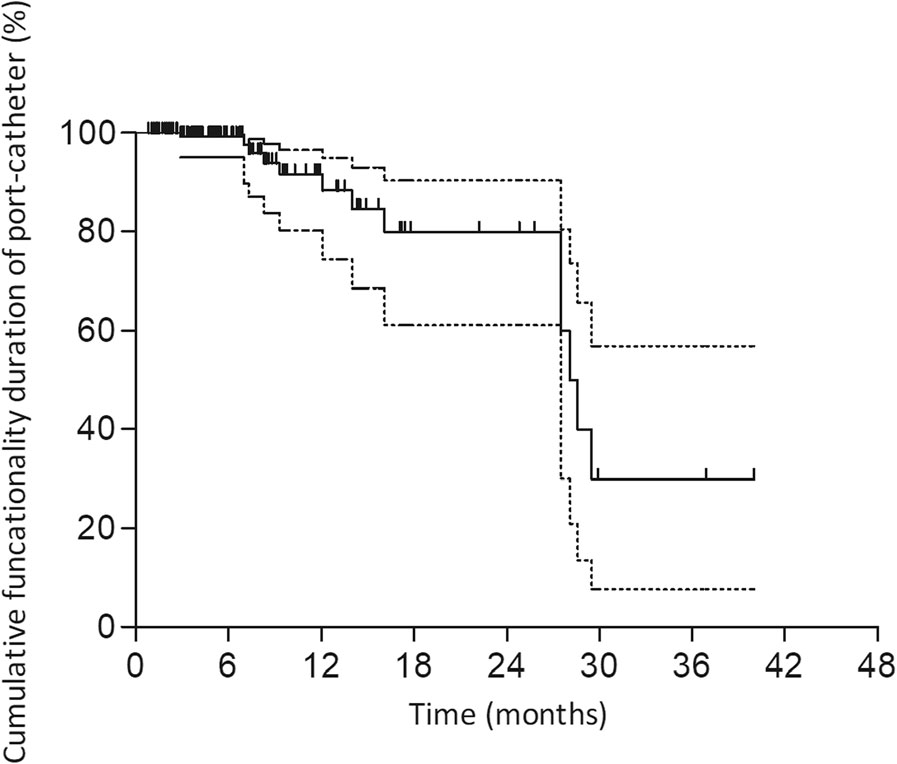
Kaplan-Meier analysis of cumulative functionality duration of port-catheter using a fixed-catheter-tip. Dotted lines represent 95% Confidence interval

Irreversible port system dysfunctions were depicted in Table 3. Predictable functionality rates of the port-catheter system at 6, 12, and 24 months were 97.5, 89.9, and 70.5%, respectively (Fig. 6).

Patients received the first course of HAIC on the second day of catheter insertion. The median number of HAIC courses was 5 (range 1–14) per patient. HAIC was discontinued with the port system due to irreversible dysfunction for 16 patients. The median number of HAIC courses in these patients with irreversible port system dysfunction was up to 8 (range 2–12). The other reasons which caused discontinuation of HAIC with port system are listed in Table 4.

**Table 4:** Reasons for Discontinuation of HAIC Treatment with port system

Reasons for Treatment Discontinuation	Numbers
Catheter irreversible dysfunction	16 (7.9%)
Catheter occlusion	7 (3.5%)
Hepatic arterial occlusion	8 (4.0%)
Catheter dislocation	1 (0.5%)
Maintenance treatment without disease progression	45 (22.3%)
Disease progression during HAIC	
Extrahepatic lesions progression	38 (18.8%)
Regrowth of hepatic lesions	32 (15.8%)
Grade 3/4 chemotherapy-related toxicity	18 (8.9%)
Rejection of treatment continuation	12 (5.9%)
Loss of follow up	16 (7.9%)
Hepatic surgery	4 (2.0%)

## Discussion

Advances in minimally invasive techniques have allowed percutaneous catheter implantation for HAIC under local anesthesia. According to a review of large published studies of percutaneous port-catheter placement for HAIC [11, 14–18, 23–25], the fixed catheter tip technique has less catheter dislocation, hepatic arterial occlusion, and high success [11, 15–18, 23]. The same results were achieved in this study, with 89.9% one-year catheter functionality and 70.5% two-year functionality, which can satisfy the HAIC treatment in clinical practice.

But in previous studies, the different embolic materials to fix the catheter tip were applied. It has been recommended in previous studies that additional use of an n-butyl cyanoacrylate (NBCA)-Lipiodol mixture along with microcoils could potentially obtain stronger fixation [11, 19]. However, Takuji’s group in a retrospective study showed that NBCA correlated with more hepatic artery obstruction (9.3%) [26], and they recommended NBCA not be used for port-catheter implantation with a fixed-catheter-tip. In this study, with coils only to fix the catheter tip, the frequency of permanent hepatic artery obstruction was lower (4.0%) when compared with previous reports with coils and NBCA fixed methods (5.4, 5.1, 9.3%) [11, 19, 26]. This may be due to lack of use NBCA.

Catheter dislocation with the coil only fixed method in this study (1%) was lower than that with coils and NBCA fixed methods (3.9, 4.4, 3.7%) [17, 19, 26]. The effect of fixing the catheter tip with coils only is likely sufficient. Less catheter dislocation in this study might be due to the less torqued catheter curve made in the aortic and more head side location access of the femoral artery.

Port catheter systems have also been developed and optimized before. Irie [24] developed a modified fixed catheter tip method with a long tapered side-hole catheter in which the indwelling catheter tip is fixed to the gastroduodenal artery with microcoils through a microcatheter coaxially advanced from the second catheter that is inserted beside the indwelling catheter exclusively for this procedure. The original intention of this modified fixed catheter tip method was to reduce mechanical stimulation to the vascular endothelium caused by the catheter. However, complications arising from the port-catheter system using an original or modified fixed catheter tip such as catheter dislocation and hepatic arterial thrombosis were not statistically significant [27]. The frequencies of catheter dislocation and hepatic artery obstruction in this study are similar to previous studies using a modified fixed catheter tip method. Besides, there were some limitations of the modified fixed catheter tip method. One being possible passage of the infused anticancer drugs over the side hole and through the open end-hole of the indwelling catheter to organs supplied with blood from the gastroduodenal or right gastroepiploic artery [28]. Reactive gastric or duodenal mucosal lesions, such as gastroduodenal ulcerations, could result from these chemotherapeutic agents [28]. Another limitation of this modified method is bilateral femoral artery puncture [24] prolonging time to ambulation and more invasive.

In term of artery access, subclavian, hypogastric, femoral, and brachial arteries have been used as percutaneous access routes to the hepatic artery in previous study [22]. In the present study, femoral artery was chosen to access of port catheter implantation because this puncture site is readily accessible and because of considerable experience of femoral punctures in the interventional radiology community. Subclavian artery access requires surgical dissection in most cases [22] and is performed through a small branch, the acromiothoracic artery. Even when subclavian access is used for catheter placement, a femoral approach is also generally required to obtain an initial angiogram and to perform embolization of the right gastric artery or a replaced hepatic artery (when present). The femoral artery access eliminates the specific complications associated with the subclavian artery access such as stroke and pneumothorax. Moreover, according to Matsumoto’s group [17], there were no significant differences between the subclavian and femoral approaches with respect to frequency of catheter dislocation. In this study, skin access site to the femoral artery was 1 cm above the inguinal skin fold and the lowest point of the catheter loop was above the level of the inguinal fold. The degree of physiologic motion of the hip joint transmitted to the tip of the catheter is potentially lesser than that with the previous transfemoral method [18, 29]. The frequency of catheter dislocation in this study was less than that in the previous transfemoral method study (1.0% vs 11%; 1.0% vs 3.9%) [15, 29].

The study have some limitations: First, it was retrospective study and the subjects were heterogeneous cancer origins. Second, there was no control group to compare the superiority of this method, because follow-up information were missing for most of the cases with non-fixed catheter tip method, which were performed in this center many years ago. Because the complications such as migration of the catheter and hepatic artery occlusion were so common, the old technique was totally abandoned in this center in the same period. Third, intrahepatic and extrahepatic perfusion information was not analyzed because CBCT hepatic angiograms were not always excellent and obvious artifacts occurred in some cases. Multiple detector computed tomography hepatic arteriogram, magnetic resonance hepatic arteriogram or SPECT were not performed to evaluate intrahepatic perfusion.

## Conclusion

In conclusion, implanting port-catheter system using percutaneous unilateral trans-femoral implantation with coil only fixed-catheter-tip method offers long-duration time functionality that can fulfill HAIC treatment for advanced liver malignancy.

## Abbreviations

5-FU: Fluorouracil
^90^Y: Yttrium-90
CBCT: Cone-beam computer tomograpfy
DEB-TACE: Drug-eluting bead transarterial chemoembolization
DSA: Digital subtraction angiography
HAIC: Hepatic arterial infusion chemotherapy
HCC: Hepatocellular carcinoma
ICC: Intrahepatic cholangiocarcinoma
NBCA: n-butyl cyanoacrylate
TACE: Transarterial chemoembolization
